# Serum NT-ProBNP/Chloride Ratio Predicts Adverse Cardiovascular Outcomes in Patients with Acute Heart Failure

**DOI:** 10.3390/biomedicines13061493

**Published:** 2025-06-18

**Authors:** Victor José Leal-Alcántara, Eder González-Macedo, Ana Cristina Maldonado-May, Alberto Santiago-Hernández, Eder Jonathan Amaro-Palomo, Sarai Hernandez-Pastrana, Anna Elisa Adib-Gracia, Rodrigo Gopar-Nieto, Daniel Sierra-Lara Martínez, José Luis Briseño-De la Cruz, Héctor González-Pacheco, Alexandra Arias-Mendoza, Diego Araiza-Garaygordobil

**Affiliations:** 1 Cardiovascular Critical Care Unit, Instituto Nacional de Cardiología “Ignacio Chávez”, Mexico City 14080, Mexico; v18leal@gmail.com (V.J.L.-A.); edergonzalezmacedo@gmail.com (E.G.-M.); dra.anamay@gmail.com (A.C.M.-M.); albertosantiagoh25@gmail.com (A.S.-H.); amaroederj@gmail.com (E.J.A.-P.); saraihpastrana@hotmail.com (S.H.-P.); annaadib1205@gmail.com (A.E.A.-G.); rodrigogopar@gmail.com (R.G.-N.); danielsierralaram@gmail.com (D.S.-L.M.); juosb@hotmail.com (J.L.B.-D.l.C.); hectorglezp@hotmail.com (H.G.-P.); aariasm@yahoo.com (A.A.-M.); 2 División de Estudios de Posgrado, Facultad de Medicina, Universidad Nacional Autónoma de México (UNAM), Campus Ciudad Universitaria, Coyoacán, Mexico City 04510, Mexico

**Keywords:** NT-proBNP, chloride, ratio, cardiovascular events, acute heart failure

## Abstract

**Background:** Heart failure (HF) is a public health issue. It represents the second most common cause of hospitalization and the leading cause in individuals over 60 years old. Tools that predict adverse outcomes in patients with HF are needed. **Objective**: This study analyzed the prognostic role of the serum NT-proBNP/chloride ratio as a predictor of major cardiovascular events in patients with acute decompensated HF. **Methods**: Patients with a confirmed diagnosis of acute decompensated heart failure were retrospectively enrolled in the study; admission NT-proBNP/chloride ratio was used to stratify patients above or below the median (>/<83). The primary composite endpoint consisted of cardiovascular mortality, decompensated HF readmission, and unplanned emergency department visits. **Results:** A total of 197 individuals were included, of whom 100 (50.7%) were classified above and 97 (49.2%) below the median. Patients showing a high ratio had a lower LVEF (31 vs. 39%), a higher proportion of previous MI (30 vs. 15%), a lower diastolic blood pressure (73 vs. 80 mmHg), and higher BUN (38 vs. 23 mg/dL) and creatinine (1.6 vs. 1.1 mg/dL). After a follow-up period of 92 ± 3 days, 46 patients (23%) presented the primary endpoint; those with a high NT-proBNP/chloride ratio showed an increased risk (HR 3.18, 95% CI 1.55–6.52, *p* = 0.0015) of the primary endpoint. After multivariate analysis, only serum NT-proBNP/chloride ratio (*p* = 0.02) and diastolic pressure (0.037) remained significant. The area under the ROC curve for the NT-proBNP/chloride ratio for predicting the primary composite endpoint was significantly superior when compared with AUC for NT-proBNP or chloride alone. **Conclusions**: The serum NT-proBNP/chloride ratio is a novel, easy to use predictor of short- and medium-term cardiovascular events in patients with acute decompensated HF.

## 1. Introduction

Heart failure is a public health issue, requiring substantial healthcare expenditure and considerably impacting quality of life, disability-adjusted life years, and mortality [1,2]. It represents the second most common cause of hospitalization and the leading cause in individuals over 60 years old, with an estimated annual cost of 108 billion dollars [3,4]. Each episode of decompensation increases the risk of in-hospital mortality, which ranges from 4% to 14.9% [5,6]. Furthermore, the risk of death persists and even increases over time, reaching 14% at 30 days and approximately 34% at one year [7].

Accurate tools that might predict adverse outcomes in patients with HF are needed. Among them, increased concentrations of N-terminal pro-B-type natriuretic peptide (NT-proBNP) correlate with increased filling pressures and ventricular dysfunction and have been associated with an increased risk of mortality and longer hospital length of stay [8]. NT-proBNP has also been shown to have a prognostic role for the prediction of adverse long-term outcomes [9,10]. Serum chloride has recently been recognized as an independent prognostic factor in HF. Although its underlying mechanisms are not fully understood, neurohumoral activation seems to be the central pathway, particularly through the renin–angiotensin–aldosterone system, acid–base imbalance, and plasma volume expansion driven by chloride-induced macula dense stimulation and chloride depletion alkalosis [11,12]. Hypochloremia has demonstrated a strong prognostic value in HF, with lower chloride levels—reflecting water and sodium retention—being inversely associated with both short- and long-term adverse outcomes. Notably, every 4.1 mEq/L decrease in serum chloride levels has been linked to a 32% increase in mortality [13,14,15].

Given the individual prognostic value of NT-proBNP and chloride, a combined biomarker such as the NT-proBNP/chloride ratio may offer enhanced predictive power for major events in HF patients.

The aim of the present study was to analyze the prognostic role of the NT-proBNP/chloride ratio in predicting major cardiovascular events in patients with decompensated heart failure.

## 2. Materials and Methods

### 2.1. Study Design Participants and Data Collection

This was a retrospective, single-center, observational study conducted at the National Institute of Cardiology Ignacio Chávez in Mexico City, between April 2018 and June 2019. The study included patients aged >18 years with either newly diagnosed or previously known heart failure (HF), who were admitted for ≥24 h to the study center, fulfilling the criteria of an HF event derived from international standardized definitions (e.g., ESC/HFA or AHA/ACC guidelines), with symptoms such as dyspnea, orthopnea, and fatigue, along with objective evidence of left ventricular dysfunction, confirmed by imaging or elevated biomarkers (NT-proBNP or BNP), and requiring urgent medical intervention or hospitalization for acute or chronic exacerbations. Patients were excluded if they lacked serum chloride or NT-proBNP measurements within the first 24 h of admission, or if their NT-proBNP level was <300 pg/mL.

Baseline clinical characteristics and clinical covariates were collected by research physicians either from the electronic medical record or directly from the imaging/laboratory tests. Laboratory data were extracted from the initial blood analyses performed upon admission, with a time window not exceeding 24 h from hospital entry. Laboratory values were measured at admission using standard hospital protocols, with NT-proBNP and BNP levels determined using commercially available immunoassay kits processed in the central laboratory at the National Institute of Cardiology. For serum chloride, measurements were obtained using standard hospital laboratory protocols.

Details concerning acute HF patients included in the analysis are as follows. Inclusion criteria: adults >18 years with heart failure, requiring ≥24 h hospitalization and meeting HF event criteria. Exclusion criteria: lack of NT-proBNP or serum chloride measurements within 24 h or NT-proBNP <300 pg/mL.

The dependent variable in this study was the NT-proBNP/chloride ratio, while the independent variables included demographic data, comorbid conditions, and clinical outcomes such as cardiovascular mortality, the need for readmission, and unplanned emergency department visits. Additionally, left ventricular ejection fraction (LVEF) and laboratory biomarkers, such as serum NT-proBNP levels and serum chloride levels, were also considered as independent variables for analysis.

Given the retrospective and observational nature of the study, written informed consent was waived. The study protocol was approved by the institutional research and ethics committee of the education department, approval number PT-18-078, and complied with the principles outlined in the Declaration of Helsinki.

### 2.2. Study Objectives

The primary objective of the study was to assess the prognostic role of the NT-proBNP/chloride ratio in the prediction of adverse cardiovascular outcomes, defined as the composite of cardiovascular mortality, the need for readmission due to acute decompensated heart failure, and unplanned emergency department visits within 120 days of the index hospitalization. The components of the primary composite outcome were based on the 2017 Cardiovascular and Stroke Endpoint Definitions for Clinical Trials [16]. The secondary outcomes included the risk of in-hospital mortality and hospital length of stay.

### 2.3. Statistical Analysis

Normality of continuous variables was assessed using the Kolmogorov–Smirnov test. Continuous variables were expressed as mean ± and standard deviation (SD) or median and interquartile range (IQR), according to their distribution, while categorical variables were presented as frequencies and percentages. Baseline characteristics were compared using the chi-square (χ^2^) test for categorical variables and the Mann–Whitney U test for continuous variables. A *p*-value <0.05 was considered statistically significant, with a 95% confidence interval applied to all comparisons. To determine the optimal cutoff point for the NT-proBNP/chloride ratio, receiver operating characteristic (ROC) curve analysis was performed to evaluate sensitivity and specificity for predicting major adverse cardiovascular events (MACE). The ratio was analyzed both as a continuous variable and dichotomized into high and low strata based on the identified cutoff for subsequent analysis: low and high serum NT-proBNP/chloride ratio, respectively.

Next, a survival analysis using a semi-quantitative approach with the Log-rank test depicted in Kaplan–Meier curves was conducted to compare the outcomes between the two groups. Subsequently, a proportional hazard–risk assessment using a Cox regression was performed to assess and estimate the association between the NT-proBNP/chloride ratio and the primary outcome. Considering the univariate analysis results, a multivariate Cox regression model was constructed, adjusting for covariates that were statistically significant, to identify variables associated with the outcome.

Finally, the discriminative performance of the NT-proBNP/chloride ratio was compared to other prognostic markers (e.g., NT-proBNP alone, serum sodium, serum chloride, and left ventricular ejection fraction) using the concordance index (C-index) and integrated discrimination improvement (IDI) to assess whether the accuracy of predicting MACE would improve with the use of the NT-proBNP/chloride ratio, compared with other prognostic indices. All statistical analyses were conducted using Stata SE version 15.1 (StataCorp, College Station, TX, USA).

### 2.4. International Review Board

This project was approved by the IRB of the study center with approval number PT-18-078, approved on 22 March 2018. Due to the retrospective nature of this study, written consent was waived.

## 3. Results

**Figure 1:**
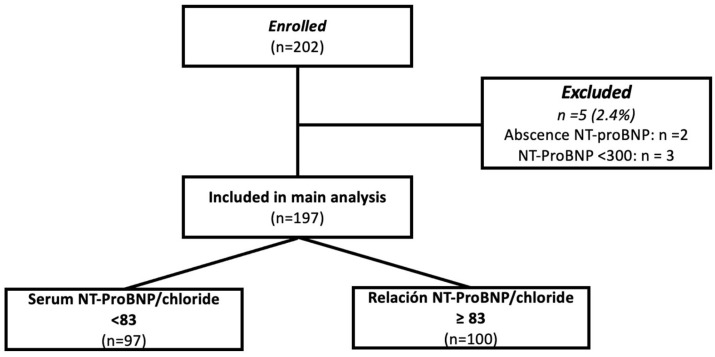
Study flow chart.

From March 2018 to February 2019, a total of 202 patients were admitted to the study center and screened for inclusion in the present study; among them, 2 had no measurement of NT-proBNP and 3 had concentrations of <300 pg/mL, and therefore were excluded. The final analytic sample was constituted by 197 patients (Figure 1). The mean age was 60 years (range: 50–74), and the proportion of women was 32% (n = 65). The mean left ventricular ejection fraction (LVEF) was 35% (range: 24–48), with 66% of the subjects having an LVEF <40%. ROC curve sensitivity/specificity analysis identified an optimal cutoff point of (>/<83); this cutoff corresponded with the median value and showed a sensitivity of 64.7% and specificity of 55.6% (LR 0.63–1.45); patients were further classified above or below the cutoff and analyzed accordingly.

In the low ratio group, there were 97 subjects (49%) with a mean age of 58 years (range: 50–67), compared to 62 years (range: 55–76) in the high ratio group (*p* = 0.109). The proportion of women was 32% in the low ratio group vs. 34% in the high ratio group (*p* = 0.762). Body mass index (BMI) was higher in the lower ratio group, 28.3 (range: 24–31), compared to 25.7 (range: 23–29) in the high ratio group. Median NT-proBNP levels were 4404 pg/mL (range: 2916–6028) in the low ratio group vs. 18,263 pg/mL (range: 11,806–25,000) in the high ratio group (*p* < 0.001). As for serum chloride levels, the mean was 101 mEq/L (range: 100–105) in the low ratio group vs. 98 mEq/L (range: 94–103) in the high ratio group (*p* < 0.001). There was also a statistically significant difference in LVEF in the univariate analysis, with a mean of 39% (range: 28–49) in the low ratio group vs. 31% (range: 20–43) in the high ratio group (*p* = 0.001). Finally, the serum NT-proBNP/ chloride ratio presented a median of 43 (range: 28–60) in the low ratio group vs. 187 (range: 124–248) in the high ratio group (*p* < 0.001). The other baseline characteristics are shown in Table 1.

### 3.1. Study Outcomes

The mean follow-up was 103 ± 4 days (range 94–111) vs. 79 ± 5 days. During this period, 46 patients (23%) experienced the composite outcome, of which 33 (71%) occurred in the high ratio group (*p* = 0.0015).

**Figure 2:**
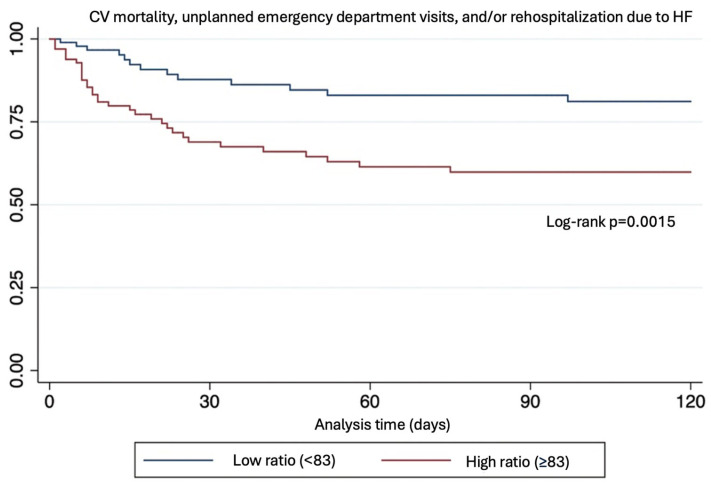
Kaplan–Mayer plot.

Patients within the high NT-proBNP/chloride ratio group had a 3-fold increase in the risk of the primary composite endpoint (OR: 3.18 [95% CI 1.55–6.52, *p* = 0.0015]). This association remained statistically significant after multivariate analysis including LVEF, age, and diabetes (Table 2 and Figure 2).

Regarding in-hospital mortality, 75% of the cases occurred in the high ratio group, with an odds ratio of 3.51 (95% CI: 1.49–8.27; *p* = 0.002). After multivariate analysis, only the serum NT-proBNP/chloride ratio (*p* = 0.02) and diastolic pressure (*p* = 0.037) predicted in-hospital mortality (*p* = 0.008), while the other variables did not. Diastolic blood pressure was shown to be inversely proportional to this outcome. Sodium (*p* = 0.59) or chloride (*p* = 0.3) by themselves did not have a significant value in this model.

### 3.2. Incremental Value of NT-ProBNP/Chloride Ratio

**Figure 3:**
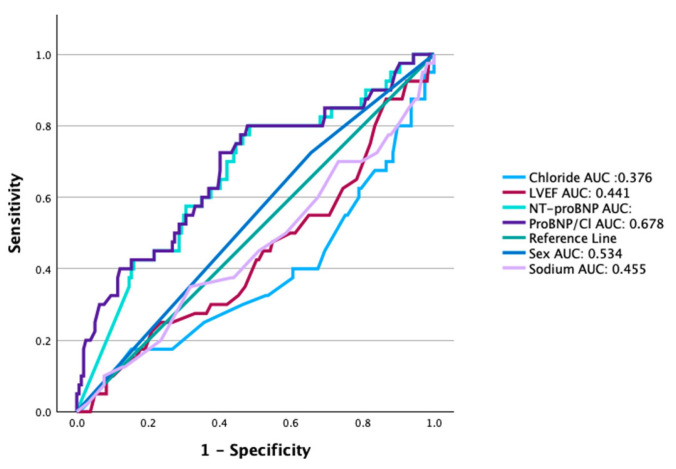
Comparison of receiver operating characteristic (ROC) curves. The area under the curve (AUC) for the NT-proBNP/chloride ratio was 0.678 (95% CI: 0.579–0.777); *p* = 0.001. NT-proBNP (0.661; 0.564–0.757), chloride (0.376; 0.271–0.480), sex (0.534; 0.436–0.633), and LVEF% (0.441; 0.338–0.543).

The area under the ROC curve for the NT-proBNP/chloride ratio for predicting the primary composite endpoint was superior (0.678, 0.579–0.777, *p* = 0.001) when compared with AUC for NT-proBNP, sodium or chloride alone, age, sex, or LVEF (Figure 3). Furthermore, the NT-proBNP/Cl index effectively reclassified the risk of outcomes in 56% of patients in whom only chloride was tested (*p* = 0.008), 47% of those in whom only sodium was analyzed (*p* = 0.004), and 25% of those in whom only NT-proBNP was considered; however, the latter was not statistically significant (*p* = 0.133).

## 4. Discussion

The present study depicts a novel and pragmatic score incorporating the NT-proBNP/chloride ratio for predicting adverse cardiovascular events in patients with a recent episode of acute decompensated heart failure. The model showed the ability to predict events combining pro-BNP and chloride in the same formula, showing an additive value above each individual component. Our model was also superior to factors such as sex, chloride, sodium, and LVEF. One of the main strengths of the study was its ability to improve integrated discrimination (IDI) or net reclassification of patients at risk of adverse outcomes when compared to the use of sodium and chloride individually, and when the performance of the NT-proBNP/chloride index was compared to NT-proBNP alone, to accurately detect MACE, the index was 25% better but without achieving statistical significance (IDI: 25%, *p* = 0.133). 

Heart failure is one of the leading causes for ED visits and presents with a wide spectrum of severity. On one hand, there are low-risk patients who do not require hospital admission; on the other hand, there are high-risk patients with a high probability of mortality or readmission to the emergency department after discharge. During the initial approach of patients with acute decompensated heart failure in the emergency department, it is crucial to identify high-risk patients who can benefit from more individualized management. Failure to identify these patients can lead to treatment delays, diagnostic procedure delays, inadequate monitoring, premature discharges, or admission to inappropriate areas, thereby increasing the risk of adverse events and wasting resources.

To support decision-making, there has been an increasing amount of information regarding the role and utility of biomarkers and scales as prognostic tools in different scenarios [17,18,19]. In 2014, Chaves demonstrated in a prospective cohort study that a BUN value of ≥43 mg/dL was the sole factor related to in-hospital mortality, while NT-proBNP levels of ≥4630 pg/dL, length of stay of ≥5 days, and BUN of ≥43 mg/dL were associated with 30-day mortality [20]. In addition to serum biomarkers, various prognostic scores are used to stratify risk in patients with acute heart failure, including the ADHERE and GWTG scales. For instance, BUN, systolic blood pressure, heart rate, and age were employed as predictive factors for in-hospital mortality within the ADHERE algorithm. Nevertheless, the information provided is confined to the intrahospital environment. Hence, a tool that predicts short- and medium-term risk would be essential for medical decision-making, like what our ratio accomplishes. Analyzing the characteristics of the ADHERE registry, we found that data used in this study represent individual hospitalization episodes rather than individual patients [21]. Multiple hospitalizations of the same patient may have been recorded as distinct entries in the registry.

The previously described scales were externally validated, demonstrating a poor ability to discriminate the risk of in-hospital mortality. The AUC for GWTG-HF was 0.57 (95% CI 0.49–0.65), and that for ADHERE was 0.58 (95% CI 0.47–0.68), while the discrimination ability for in-hospital and 4-month risk in our ratio was higher: 0.65 (0.56–0.75, *p* = 0.03) [19].

These scales described before include many variables related to the pathophysiology of heart failure. However, some of them may be more useful, such as NT-proBNP, which has demonstrated in multiple studies its value as a prognostic factor in risk discrimination for patients with acute decompensated heart failure. In 2016, Sprockel described that by incorporating NT-proBNP levels into these scales, the ability to discriminate risk can be substantially improved [22].

Recently, the role of serum chloride (Cl) as a prognostic marker and its implications in heart failure has taken attention, and some studies suggest that hypochloremia may be a stronger prognostic marker than age, left ventricular ejection fraction (LVEF), or NT-proBNP for predicting mortality or all-cause hospitalization. Although pathophysiological mechanisms underlying hypochloremia are complex, it is well established that this electrolyte imbalance triggers significant neurohumoral activation, which negatively affects the cardiovascular system [23]. One potential mechanism may involve the exacerbation of congestion and the increased pressure within the cardiac chambers. This, in turn, could contribute to the worsening of heart failure symptoms, as the body attempts to compensate for fluid overload through neurohormonal pathways, further impacting outcomes.

Additionally, an inverse relationship between chloride levels and NT-proBNP has been observed, suggesting a synergistic effect between these markers in the context of heart failure. While NT-proBNP is a well-established indicator of heart failure severity and volume overload, chloride may reflect a distinct aspect of the pathophysiological process, such as impaired renal function or severe congestion, which is less directly captured by NT-proBNP levels alone [24]. The combination of these variables (chloride and NT-proBNP) has shown independent prognostic value, and their integration into a predictive model is a novel aspect of this study, offering valuable insights into heart failure prognosis that have not been fully explored before.

We provided data showing that the ratio previously mentioned is directly associated with adverse events, independently if there is a preserved or reduced LVEF, as published in other registries with similar baseline characteristics, although most of our patients had the last one. Overall, we had a cardiovascular event frequency of 23%, which is similar to that described by Gonzalez-Pacheco in a cohort alike to our own [6]. In addition to the risk of cardiovascular events with the NT-proBNP/chloride ratio, multivariate analysis showed an association with diastolic blood pressure. The relationship between low diastolic pressure and cardiovascular events is well established. Tsujimoto (2018) reported that compared with DBP 80–89 mmHg, patients with DBP <70 mmHg had a higher frequency of adverse outcomes, which resulted in even a greater number for those with a DBP <60 mmHg (RR: 2.19–95% CI 1.72–2.78) [25]. Although the mechanisms by which this condition affects prognosis are not well established, a possible explanation is the reduction in myocardial blood flow, due to a decrease in coronary perfusion pressure, as discussed by McEvoy (2016) [26].

As is known, NT-proBNP levels can vary according to different pathologies. Obesity is a factor that inversely impacts these figures. In this analysis, the mean BMI was 27, while 20.8% were found to have some degree of obesity. Furthermore, creatinine levels were heterogeneous, with a mean of 1.4 mg/dL (IQR 0.55–1.85), which also results in biomarker fluctuations. These conditions are common in the context of a patient with decompensated heart failure and reported in multiple research projects; however, care should be taken when interpreting the results with these entities and consider that if another specific population is evaluated, the results may be different [27,28,29].

There are limitations to this study, including the sample size. It is important to note that the design was retrospective, and, as such, it carries a risk of selection and information bias. In addition, the lack of detailed information regarding medication doses and adherence to treatment was the reason these elements were not included in the analysis. While pharmacological treatment is an important factor in heart failure management, its absence in our analysis does not allow us to assess its potential influence on the NT-proBNP/chloride ratio and patient outcomes. Further prospective studies are needed to integrate these factors into the model. The median follow-up was 92 ± 3 days, and different outcomes may be observed in a long-term evaluation. Future studies will include longer follow-up periods to better assess the long-term prognostic value of the NT-proBNP/chloride ratio in heart failure patients. Additionally, this investigation only included patients from a single center, so the results may not be generalizable, and external validation is required. Moreover, different local protocols, healthcare access, and availability of resources might impact on the way the ratio can be applied. The NT-proBNP/chloride ratio is a useful tool for the early identification of subjects with decompensated heart failure at higher risk of adverse cardiovascular outcomes, both during hospitalization and in the short term. This is a novel and practical instrument that can be integrated into the assessment of patients with this condition, given its greater predictive capacity compared to other factors. This study is the first of its kind, so further research and long-term follow-up in prospective studies are necessary for validation.

## 5. Conclusions

Our study suggests that the serum NT-proBNP/chloride ratio may be useful as a tool for predicting short- and medium-term cardiovascular outcomes in patients with heart failure and a recent event of acute decompensation. Additionally, the serum NT-proBNP/chloride ratio can predict a higher risk of in-hospital mortality. This instrument could become a strategy to address the significant challenge in identifying individuals at high risk of experiencing adverse cardiovascular events and implementing measures to reduce the burden of the disease.

## Figures and Tables

**Table 1:** Baseline characteristics.

Baseline Characteristics by Group			
	Low NT-proBNP/Cl Ratio (<83) [n = 97]	High NT-proBNP Ratio (≥83) [n = 100]	*p* Value
Demographics			
Main age, years	58 (50–67)	62 (55–76)	0.109
Women (%)	31 (32)	34 (34)	0.762
Men (%)	66 (68)	66 (66)	0.762
BMI (IQR)	28.39 (24–31)	25.74 (23–28)	0.004
Comorbilities			
HAS (%)	55 (57)	45 (45)	0.102
DM (%)	40 (41)	36 (36)	0.457
LVFE % (IQR)	39 (28–49)	31 (20–43)	0.001
Smoking (%)	42 (43%)	30 (30%)	0.053
History of myocardial infarction (%)	15 (15%)	30 (30%)	0.015
History of cancer (%)	3 (3%)	1 (1%)	0.3
History of stroke (%)	3 (3%)	1 (1%)	0.3
Admission vital signs			
Heart rate bpm (RIC)	93 (77–102)	86 (67–103)	0.073
Systolic blood pressure, mmHg (IQR)	125 (110–140)	119 (100–135)	0.212
Diastolic blood pressure, mmHg (IQR)	80 (70–90)	73 (60–86)	0.004
Respiratory rate, bpm (IQR)	20 (16–21)	21 (18–24)	0.14
Oxygen saturation, % (IQR)	89 (89–94)	89 (88–92)	0.685
Admission laboratories			
Hemoglobine g/dL (IQR)	14.4 (13.4–15.9)	13.7 (12.3–15.4)	0.026
Hematocrit % (IQR)	43 (40–47)	42 (31–46)	0.099
Leucocites 10^3/mcL (IQR)	10.7 (7.8–12.9)	9.7 (6.7–11.4)	0.117
Platelets 10^3/mcL (IQR)	228 (175–259)	208 (163–254)	0.016
Glucose mg/dL (IQR)	181 (105–229)	148 (102–161)	<0.001
Blood Urea Nitrogen (IQR)	23 (15–27)	38 (21–52)	<0.001
Creatinine mg/dL (IQR)	1.1 (0.8–1.3)	1.6 (1.0–2.0)	<0.001
Sodium mmol/L (IQR)	134 (132–138)	132 (129–136)	0.001
Potassium mmol/L (IQR)	4.1 (3.9–4.4)	4.3 (3.9–4.8)	0.032
Chloride mmol/L (IQR)	101 (100–105)	98 (94–103)	<0.001
NT-proBNP pg/mL (IQR)	4404 (2916–6028)	18,263 (11,806–25,000)	<0.001
Serum NT-proBNP/chloride ratio	43 (28–60)	187 (124–248)	<0.001
Albumin g/dL (IQR)	3.3 (3.0–3.7)	3.1 (2.8–3.5)	0.002

IQR: interquartile range.

**Table 2:** Risk estimation for evaluated outcomes and risk predictor estimator.

Risk Estimation for Outcome Prediction			
Group	*p* Value	Hazard Ratio	95% Confidence Interval (Lower–Higher)
CV mortality, rehospitalization for HF and/or visits to the emergency room (%)	0.0015	3.18	1.55–5.52
In-hospital mortality	0.002	3.51	1.492–8.275
Length of stay	0.214	1.47	0.80–2.70

CV: cardiovascular; HF: heart failure.

## Data Availability

All important and relevant data are included in the Methods and Results sections. If any additional data are required, please contact the corresponding author.
